# Improving Model Performance on the Stratification of Breast Cancer Patients by Integrating Multiscale Genomic Features

**DOI:** 10.1155/2020/1475368

**Published:** 2020-08-25

**Authors:** Yingyi Hao, Li He, Yifan Zhou, Yiru Zhao, Menglong Li, Runyu Jing, Zhining Wen

**Affiliations:** ^1^College of Chemistry, Sichuan University, Chengdu, Sichuan 610064, China; ^2^Biogas Appliance Quality Supervision and Inspection Center, Biogas Institute of Ministry of Agriculture, Chengdu, Sichuan 610041, China; ^3^College of Computer Science, Sichuan University, Chengdu, Sichuan 610064, China; ^4^College of Cybersecurity, Sichuan University, Chengdu, Sichuan 610064, China; ^5^Medical Big Data Center, Sichuan University, Chengdu, Sichuan 610064, China

## Abstract

In clinical cancer research, it is a hot topic on how to accurately stratify patients based on genomic data. With the development of next-generation sequencing technology, more and more types of genomic features, such as mRNA expression level, can be used to distinguish cancer patients. Previous studies commonly stratified patients by using a single type of genomic features, which can only reflect one aspect of the cancer. In fact, multiscale genomic features will provide more information and may be helpful for clinical prediction. In addition, most of the conventional machine learning algorithms use a handcrafted gene set as features to construct models, which is generally selected by a statistical method with an arbitrary cut-off, e.g., *p* value < 0.05. The genes in the gene set are not necessarily related to the cancer and will make the model unreliable. Therefore, in our study, we thoroughly investigated the performance of different machine learning methods on stratifying breast cancer patients with a single type of genomic features. Then, we proposed a strategy, which can take into account the degree of correlation between genes and cancer patients, to identify the features from mRNAs and microRNAs, and evaluated the performance of the models with the new combined features of the multiscale genomic features. The results showed that, compared with the models constructed with a single type of features, the models with the multiscale genomic features generated by our proposed method achieved better performance on stratifying the ER status of breast cancer patients. Moreover, we found that the identified multiscale genomic features were closely related to the cancer by gene set enrichment analysis, indicating that our proposed strategy can well reflect the biological relevance of the genes to breast cancer. In conclusion, modelling with multiscale genomic features closely related to the cancer not only can guarantee the prediction performance of the models but also can effectively provide candidate genes for interpreting the mechanisms of cancer.

## 1. Introduction

Compared with the microarray technology, next-generation sequencing technology including DNA sequencing [1, 2] and RNA sequencing [3, 4] provides multiscale genomic features, such as mRNA expression [5, 6], microRNA expression [7, 8], and gene structure variation [9, 10], to characterize cancers in different aspects at the molecular level. These features had been widely used to construct models in clinical cancer researches for distinguishing the subtypes of cancer [11] and stratifying the patients [12], as well as predicting the prognosis of cancers [13]. The genes used in the models are commonly applied to the subsequent interpretation of the mechanisms of cancers.

Generally, the whole modeling procedure mainly includes the gene selection and the parameter estimation of prediction models. The clinical cancer samples were firstly divided into two groups according to the phenotypic status. By comparing the phenotypic differences between the two groups of samples, a set of genes can be identified by a statistical method with an arbitrary cut-off. In previous studies, only the single type of genomic features, including mRNA expression level [14], microRNA expression level [15], and gene mutation status [16], as well as the copy number variations [17, 18], was applied to characterizing the phenotypic difference of the samples. Considering the fact that a single type of genomic features can only reflect one aspect of the characteristics of tumor samples, multiscale types of features should be able to better represent the clinical status of the samples. However, there are few studies on how to use multiscale features to improve the prediction performance of the clinical models [19], which needs to be further explored. In addition, the gene selection process using statistical methods mainly focuses on the differences between the two groups of samples and cannot ensure the causal relationship between the differences and the predicted endpoint. A certain number of false positive genes will be involved in the predictive models. Meanwhile, an arbitrary cut-off used in the statistical method will also lead to the neglect of some important disease-related genes. As a result, the reliability of models based on limited data sets will be reduced.

Therefore, to explore the benefits of integrating multiscale genomic features for clinical prediction, we thoroughly investigated the performance of models constructed with a single type of genomic features, and as a comparison, we constructed models with multiscale features to predict the same clinical endpoints. Considering that the performance of the models is mainly related to the predictability of clinical endpoints [20], we evaluated the model performance by using the same endpoint, namely, the estrogen status of breast cancer patients. In our study, the patients were categorized into two groups according to their estrogen status: estrogen positive (ER+) and estrogen negative (ER-). Using two types of genomic features separately, namely, mRNA expression level and microRNA expression level, we constructed the models with 20 popular machine learning algorithms and five feature selection methods. In total, over 500 models were constructed and evaluated in this study. For the comparison, we used both the mRNA expression level and the microRNA expression level as features to construct models and stratify the patients. In addition, for the purpose of generating the features of biological relevance to breast cancer, we proposed a strategy by using the Shapley additive explanation (SHAP) method [21] to screen the mRNAs and microRNAs. Our results showed that, compared with the models with a single feature type, the performance of the models on stratifying the breast cancer patients was improved effectively by combining the two feature types of the mRNA expression level and the microRNA expression level. The results of gene set enrichment analysis indicated that mRNAs and microRNAs identified by our proposed strategy had strong correlation with breast cancer. Our proposed strategy was also applied to the stratification of the patients in kidney renal clear cell carcinoma (KIRC) and thyroid carcinoma (THCA) data sets. The mean MCCs for KIRC and THCA achieved by the models with selected multiscale features were 0.512 and 0.447, respectively, indicating that the model using the mRNAs and microRNAs closely related to the cancers can not only ensure the prediction performance but also effectively reduce the number of features and avoid overfitting, which should ensure its reliability in future prediction.

## 2. Materials and Methods

### 2.1. Data Set

The mRNA and microRNA sequencing data of breast invasive carcinoma (BRCA) samples as well as the clinical information were downloaded from the NCI's Genomic Data Commons (GDC) [22], which included 1092 RNA sequencing samples and 1079 microRNA sequencing samples. We took the intersection of the samples and finally kept 1025 samples with both the mRNA expression and microRNA expression profiles for the subsequent modeling. In the modeling procedure, 80% samples (819 samples) were randomly selected as the training set to construct the models and the rest of the samples (206 samples) were used as the independent test set to validate the models. According to their estrogen receptor (ER) status in clinical information, the samples were categorized in either the ER positive group or the ER negative group. Each sample contained the expression values of 60,482 mRNAs and 1886 microRNAs, which were summarized in the form of fragments per kilobase of exon per million fragments mapped (FPKM). The expression values of mRNA and miRNA were separately normalized. By comparing the expression values of mRNAs and microRNAs of the samples between the two ER groups in the training set with Student's *t*-test, 24,585 mRNAs and 722 microRNAs for each sample, in total, were kept as features by using a cut-off of *p* < 0.05. In addition, to validate the performance of the proposed strategy, we also applied it to stratifying the patients in KIRC and THCA data sets, which included 588 and 567 patients, respectively. The patients with tumor stages III and IV were categorized into the high-risk group, and the rest of the patients were categorized into the low-risk group. We used the same modeling procedure that had been used in the BRCA data set to stratify the patients.

### 2.2. Algorithms for Feature Selection

Five commonly used feature selection methods, namely, *Fold Change* (*FC*), *OneR* [23], *ReliefF* [24], *InfoGain* [25], and *GainRatio* [26], were involved in this study. All the methods can rank the mRNAs and microRNAs by the calculation results, and the top 30, 50, 100, 200, 300, and 500 of the mRNAs and microRNAs were separately extracted as features to construct the models. For the method of *FC*, the log 2-tranformed fold change for each mRNA or microRNA between the samples in the ER positive and ER negative groups was calculated and used for ranking the features. When extracting the top *n* features, we separately took the top *n*/2 features from upregulated and downregulated directions. *OneR* separately used each feature to classify the samples and calculated the error rate and then assigned a weight to the feature according to the error rate. As for *ReliefF*, it randomly selected a sample from the training set as well as its *k* nearest neighbors of the same and different classes. By calculating the differences of features between the two classes of samples, each feature was assigned a weight. The method updated the weights continuously through multiple resampling. Both *InfoGain* and *GainRatio* were derived from information theory and assigned the weights to the features according to their information entropies [27]. *GainRatio* can be considered as a normalization of *InfoGain*, but the information entropies of the features calculated by *GainRatio* were totally different from those calculated by *InfoGain*. In the end, we ranked the features by their weights and selected the top *n* features for modeling.

### 2.3. Machine Learning Methods for Modeling

To investigate the model performance with a single type of genomic features, 20 modeling methods, including ten ensemble learning methods (*AdaBoostM1*, *Dagging*, *LogitBoost*, *RandomCommittee*, *RandomForest*, *RotationForest*, *MultiClassClassifier*, *RandomSubSpace*, *Decorate*, and *MultiBoostAB*) and ten single classifiers (*SimpleLogistic*, *NaiveBayes*, *LibSVM*, *LocalKnn*, *LibLINEAR*, *Hoeffding Tree*, *DecisionTable*, *CSForest*, *ADTree*, and *Ridor*), were involved in our study. For each modeling method, we evaluated the performance of the models, which were separately constructed by using the expression levels of the top *n* mRNAs and microRNAs identified by the five feature selection methods. For the purpose of comparison, all the models were constructed by directly using the script interface of WEKA (version 3.8.4) [28] with default parameters. The names of the methods in this study directly came from their names used in WEKA. Three performance metrics, namely, accuracy (ACC), Matthews's correlation coefficient (MCC), and F1 score, were applied to the model evaluation, which were defined by the following:

(1) $$\begin{array}{c} \text{Accuracy }\left(\text{ACC}\right)=\frac{\text{TP}+\text{TN}}{\text{TP}+\text{TN}+\text{FP}+\text{FN}}, \end{array}$$

(2) $$\begin{array}{c} \text{MCC}=\frac{\text{TP}\times \text{TN}-\text{FP}\times \text{FN}}{\sqrt{\left(\text{TP}+\text{FP}\right)\left(\text{TP}+\text{FN}\right)\left(\text{TN}+\text{FP}\right)\left(\text{TN}+\text{FN}\right)}}, \end{array}$$

(3) $$\begin{array}{c} \text{F}1=\frac{2\text{TP}}{2\text{TP}+\text{FP}+\text{FN}}, \end{array}$$

where TP, TN, FP, and FN indicate the numbers of true positives, true negatives, false positives, and false negatives, respectively.

### 2.4. Shapley Additive Explanation Method

The Shapley value was firstly proposed by Shapely based on game theory [29], which was raised for measuring the contribution from every player in cooperation. For the past ten years, the Shapely value has been utilized for explaining the modeling results from machine learning methods [30, 31]. Recently, a python module named SHAP (SHapley Additive exPlanations) for generating the Shapely values from machine learning models was released at http://github.com/slundberg/shap [32], and this module was used in this work as the explainer of random forest. As a brief introduction, the Shapley values could be used for measuring the impact to the built model from the following feature:

(4) $$\begin{array}{c} \phi_{i}\left(f,x\right)=\underset{S\subseteq S_{\text{all}/\left\{i\right\}}}{\sum }\frac{\left|S\right|!\left(M-\left|S\right|-1\right)!}{M!}\left[f_{x}\left(S\cup \left\{i\right\}\right)-f_{x}\left(S\right)\right]=\underset{S\subseteq S_{\text{all}/\left\{i\right\}}}{\sum }\frac{1}{C_{M}^{\left|S\right|}\left(M-\left|S\right|\right)}\left[f_{x}\left(S\cup \left\{i\right\}\right)-f_{x}\left(S\right)\right], \end{array}$$

where *M* is the number of features, *S* is a subset of the features, *f* is the model, *S*_all/{*i*}_ represents all the possible subsets that exclude feature *i*, and *f*_*x*_ is the conditional expectation function. With the Shapley values, a model can be represented as a linear combination of the Shapely values:

(5) $$\begin{array}{c} f\left(x\right)=\phi_{0}\left(f\right)+\overset{M}{\underset{i=1}{\sum }}\phi_{i}\left(f,x\right), \end{array}$$

where *ϕ*_0_(*f*) = *f*_*x*_(∅). The implementation of the Shapley value is complicated since there would be too many terms for evaluating, and usually only a few approximate ways are used [33].

In this work, to generate the Shapley values, the data set was firstly trained by a random forest classifier from the SKlearn package. Then, the tree explainer was generated from the trained model. The Shapley values *S*_*x*,*y*_ could be generated by the explainer, where *x* and *y* are the index of samples and features. The Shapley values could be different according to the samples, but our target is to find out the feature which could influence the samples as much as possible. Therefore, a 10-fold crossvalidation strategy was used to select the features: (1) The data sets were divided into 10 folds, and every fold could be the test data set and the others as the training data sets. (2) For every fold, the training set was used to generate the Shapley values *S*_*x*,*y*_^*k*^(*k* = 1, ⋯, 10), then the importance of features was acquired by computing the summation of the absolute values:

(6) $$\begin{array}{c} {S\text{sub}}_{y}^{k}=\underset{x}{\sum }\left|S_{x,y}^{k}\right|. \end{array}$$

Since there will be lots of values in every *S*sub_*y*_^*f*^, we filtered *S*sub_*y*_^*f*^ by keeping the top 300 maximum values (i.e., set the top 300 maximum number as 1 and the others as 0). Then, the overall feature importance could be generated by computing the summation:

(7) $$\begin{array}{c} F_{y}=\underset{k}{\sum }{\tilde{S\text{sub}}}_{y}^{k}, \end{array}$$

where ${\tilde{S\text{sub}}}_{y}^{k}$ is the filtered *S*sub_*y*_^*k*^, and all the features which had *F*_*y*_ larger than 5 were picked out for subsequent modeling. In this study, the selected feature was ranked in the top 300 among those which were more than 6 folds.

## 3. Results

### 3.1. Study Design

**Figure 1:**
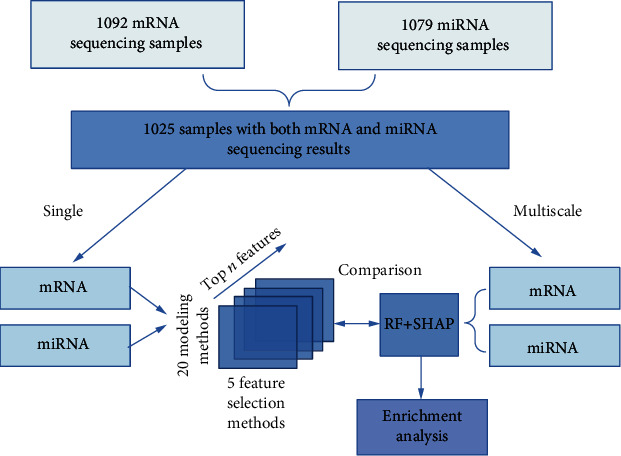
The workflow of our study.

In this study, the mRNA sequencing and microRNA sequencing data of 1025 BRCA samples were used to investigate predictive models with single type and multiple types of genomic features. The BRCA samples were stratified into the ER positive and ER negative groups according their ER status. Using the expression levels of mRNAs and microRNAs, we separately applied 20 machine learning algorithms with five feature selection methods to construct models for the stratification of BRCA samples. As a comparison, the combined features including expression levels of mRNAs and microRNAs were also used to build the models. For the purpose of obtaining the interpretable features, we proposed the SHAP method to identify the genomic features which were closely related to the cancer. The selected mRNAs and microRNAs were analyzed by gene set enrichment analysis. The entire workflow is depicted in Figure 1.

### 3.2. Model Performance with a Single Type of Genomic Features

**Figure 2:**
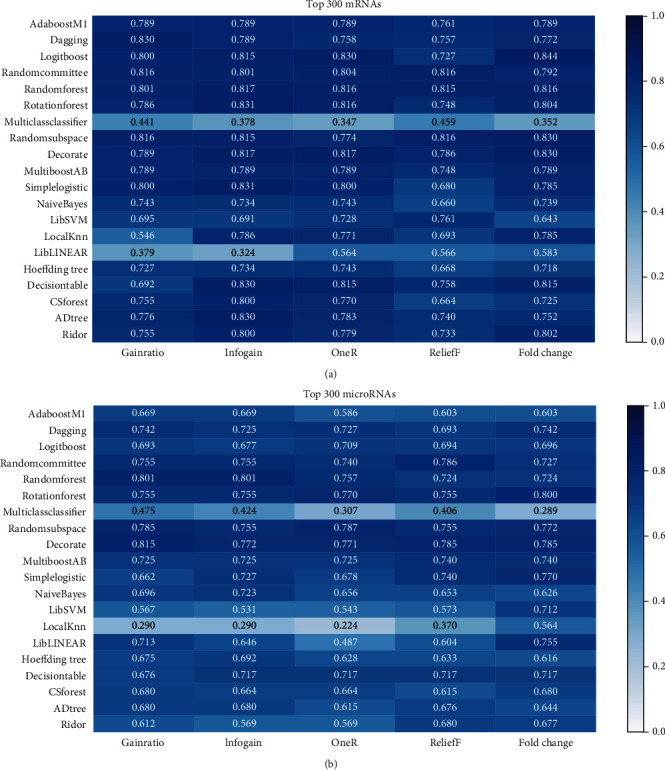
The MCCs achieved by different machine learning algorithms combined with different feature selection methods. (a) Prediction results with the top 300 mRNAs as features. (b) Prediction results with the top 300 microRNAs as features.

To evaluate the model performance, the top *n* mRNAs and microRNAs (*n* = 30, 50, 100, 200, 300, and 500) were separately selected by five feature selection methods and used as features for 20 modeling algorithms.  Figure 2 shows the prediction results of the models with the top 300 mRNAs and microRNAs as features. The results of the predictive models using the top 30, 50, 100, 200, and 500 features are exhibited in Supplementary Figure 1. In general, the performance of models using the expression levels of mRNAs was better than that using the expression levels of microRNAs. Additionally, there was a slight difference in the model performance when using a different number of top ranked features for modeling (Supplementary Figure 2).

**Figure 3:**
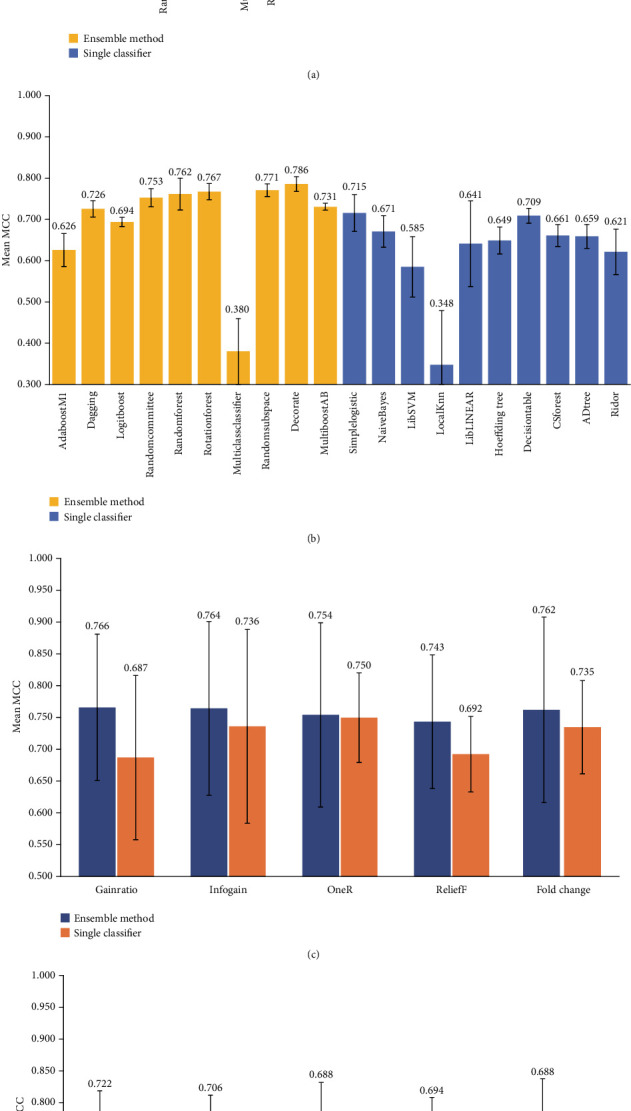
Mean MCCs achieved by the models across different machine learning algorithms and different feature selection methods. (a) Mean MCCs achieved by different machine learning algorithms with the top 300 mRNAs as features. (b) Mean MCCs achieved by different machine learning algorithms with the top 300 microRNAs as features. (c) Mean MCCs achieved by using different feature selection methods with the top 300 mRNAs as features. (d) Mean MCCs achieved by using different feature selection methods with the top 300 microRNAs as features.

The mean MCCs across different machine learning methods and different feature selection methods are shown in Figure 3. From the figure, it can be seen that the mean MCCs achieved by all the machine learning methods except *MultiClassClassifier* and *LibLINEAR* with mRNAs as features were greater than 0.700 (Figure 3(a)). For the machine learning methods with microRNAs as features, the MCC values achieved by more than half of the methods were less than 0.7 (Figure 3(b)). It indicated that the information carried by the expression levels of mRNAs might be more suitable than those of microRNAs for constructing the models to stratify the ER samples. For the same type of genomic features, the performance of ensemble modeling methods was superior to that of single modeling methods. Especially for microRNA, the mean MCCs achieved by seven out of ten ensemble methods were greater than 0.700, while only the MCCs achieved by *SimpleLogistic* and *DecisionTable* in the ten single modeling methods were higher than 0.700. Among all the machine learning methods, random forest performed the best (mean MCC = 0.813) by using the expression levels of mRNAs as features.

As for the feature selection methods, there was no significant difference in the mean MCC values between five feature selection methods when using mRNAs as features to construct the ensemble predictive models (Figure 3(c)), which performed the best among all the models. The main difference in mean MCCs was whether to use mRNAs or miRNAs as features for prediction. By using mRNAs as features for modeling, the mean MCCs were generally higher than those achieved by the models using miRNAs as features (Figures 3(c) and 3(d)).

### 3.3. Model Performance with Multiscale Genomic Features

**Figure 4:**
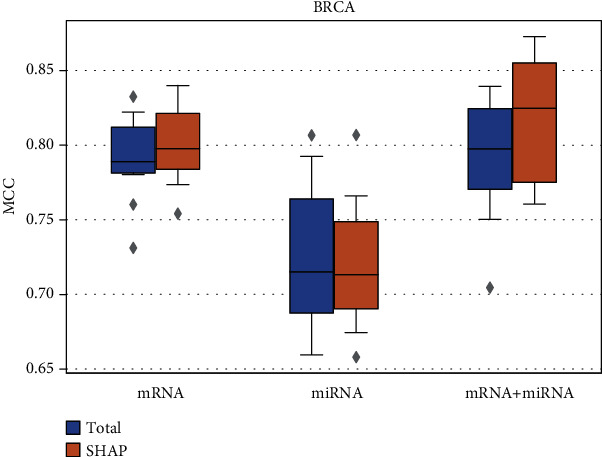
MCCs for the independent test set by using mRNAs, microRNAs, and the combination of mRNAs and microRNAs as features. The Welch *t*-test *p* value for the MCCs achieved by the models with combined mRNAs and miRNAs before and after feature selection was 0.171.

To demonstrate the importance of selecting interpretable genomic features for modeling, we applied SHAP to generate the features from mRNAs and microRNAs, as well as from multiscale genomic features, which are an integration of mRNAs and microRNAs in this study. As a comparison, we separately constructed the models with all the features of mRNAs, microRNAs, and the multiscale genomic features. The random forest algorithm, which performed best in the previous step, was applied for modeling. The prediction results for the independent test set are shown in Figure 4. The MCCs, ACCs, and F1s for the independent test set are listed in Supplementary Table 1.

If all the mRNAs were used as features, the model performance (mean MCC = 0.791) was slightly worse than the best model in the previous step (mean MCC = 0.813). It indicated that, for the same modeling method, the feature selection process can help remove the useless variables and improve the prediction performance of the model to some degree. Then, we proposed SHAP to rank the features and took the top 300 features for modeling. For the single type of genomic features, we finally identified 289 mRNAs and 291 microRNAs from the total features of 24,585 mRNAs and 722 microRNAs, respectively. From the multiscale feature set, we identified 290 genomic features including 283 mRNAs and 7 microRNAs. After the feature selection procedure, the performance of the model constructed with mRNAs as features was improved (median MCC = 0.798). Similarly, the median MCC achieved by the model with multiscale genomic features was 0.825, which was higher than that achieved by the model before feature selection (median MCC = 0.798). Interestingly, after feature selection, the prediction performance of the model with mRNA as a feature is close to that of the model with multiscale genomic features. We inspected the selected features in the list of multiscale genomic features and found that only seven out of 290 features were microRNAs and the rest were mRNAs. It indicated that the contribution of multiscale genomic features was dominated by the expression levels of mRNAs, which might be more related to the phenotypic difference of the samples. As for the microRNAs, the performance of the model was the worst when it was used for predicting the ER status, regardless of whether feature selection was conducted or not. Our proposed strategy and the same modeling procedure were also applied to the KIRC and THCA data sets. The prediction results for KIRC and THCA are shown in Supplementary Figures 3 and 4 and listed in Supplementary Tables 2 and 3. The median MCCs for stratifying the patients in the KIRC and THCA data sets by the models with multiscale features after feature selection were 0.514 and 0.438, respectively (Supplementary Figures 3 and 4).

### 3.4. Gene Function Analysis

To elucidate the biological relevance of the selected genomic features to the cancers, the gene set enrichment analysis was conducted in the subsequent analysis. The KEGG pathways enriched by using DAVID (https://david.ncifcrf.gov/) [34] with 289 mRNAs are listed in Table 1. Among four significantly enriched pathways, two of them named *Pathways in cancer* (*p* = 0.026) and *MicroRNAs in cancer* (*p* = 0.036) were directly associated with cancers. The top ten enriched Gene Ontology (GO) terms that related to the biological process and molecular functions of the genes are listed in Tables 2 and 3, respectively. From the biological process, we found that the gene set was significantly associated with the homeostasis in cells (*GO:0042592~homeostatic process* and *GO:0048871~multicellular organismal homeostasis*) and the response to drugs (*GO:0042493~response to drug*). In addition, the gene set was also related to the regulation of hormones (*GO:0010817~regulation of hormone levels*). As for the molecular functions of the genes, GO terms related to protein binding and enzyme activity were significantly enriched. Our results suggested that the mRNAs identified by our strategy are closely associated with the mechanism of cancers.

For the enrichment analysis of 291 microRNAs, it was conducted by using GeneCodis 4.0 (https://genecodis.genyo.es/) [35], which is a web-based tool for providing concurrent annotations of a gene set. The enriched KEGG pathways and GO terms related to biological processes and molecular functions are listed in Supplementary Tables 4, 5, and 6, respectively. Only one pathway named *MicroRNAs in cancer* was significantly enriched, indicating that the microRNAs identified by our strategy were mainly associated with cancer. The top 10 enriched GO terms of biological processes indicated that the 291 microRNAs were significantly associated with gene silencing (*GO:0035195~gene silencing by miRNA*) and inhibition of translation (*GO:0035278~miRNA mediated inhibition of translation*). Most of the top 10 GO terms referred to the negative regulation of genes. The enriched GO terms of molecular functions reflected that the functions of 291 microRNAs were correlated with DNA and RNA binding.

In addition, only seven microRNAs were involved in the final decided multiscale genomic feature set. Four of them, namely, miR-135b, miR-190b, miR-224, and miR-588, had been reported to be associated with various cancers. miR-135b is closely related to cervical cancer, lung cancer, and prostate cancer. It is commonly upregulated in cervical cancer cell lines, and the downregulation of miR-135b can inhibit the growth of cervical cancer cells [36]. It can also promote the metastasis of lung cancer by regulating *LZTS1* and multiple targets in the Hippo pathway [37] and inhibiting the metastasis of prostate cancer by targeting *STAT6* [38]. miR-190b is significantly upregulated in hepatocellular carcinoma cells and interacts with the 3′-untranslated region of *IGF-1* [39]. Both miR-224 and miR-588 showed clear evidence of being associated with breast cancer. miR-224 plays an important role in preventing the metastasis of breast cancer cells to bone by directly inhibiting tumor suppressor gene *RKIP* [40]. miR-588 is considered as an important prognostic biomarker in breast cancer because it is significantly downregulated in breast cancer cells and its abnormal expression level has been reported to be closely associated with the poor prognosis of breast cancer patients [41].

## 4. Discussions

For the studies in tumorigenesis, the purpose of modeling is not only to accurately predict the prognosis of cancer patients but also to identify genes with significant biological relevance to cancers. Considering that most of the existing modeling processes involve steps of handcrafted gene selection, it is possible to neglect cancer-related genes or to include false positive genes in the selected gene set, which will interfere with the subsequent biological analysis. Therefore, in this study, we thoroughly conducted a comparative study on the model performance with different genomic features and different modeling methods and proposed a strategy to identify the disease-related genes. All the models were tested by using the mRNA and microRNA sequencing data of BRCA samples. According to the ER status of the patients, the samples were categorized into the ER positive and ER negative groups, which were used as the clinical endpoint for prediction.

The model performance mainly depended on which type of genomic features was used for modeling. The models with the expression levels of mRNAs as features showed better performance than those with the expression levels of microRNAs (Figures 2 and 3), indicating that the expression profile of mRNAs might be more related to the phenotypic difference of BCRA samples. Compared with the best model constructed with a single type of genomic features (mean MCC = 0.813), the model performance was further improved when using multiscale genomic features for modeling (mean MCC = 0.819). It has been suggested that integrating the information of the expression profiles of mRNAs and microRNAs can be helpful for ER status prediction. It is worth noting that mRNAs still accounted for a large proportion of the final identified multiscale genomic features. When using the same type of genomic features for modeling, the ensemble modeling methods showed better prediction performance (Figure 3). Random forest with mRNAs as features performed the best among all the models (mean MCC = 0.813). As for the feature selection methods, no matter what method was used, there was only a slight difference in the performance of the models (Figure 3).

When applying our strategy to the genomic feature selection, 289 mRNAs and 291 microRNAs were selected from the total features of mRNAs and microRNAs, respectively. The results showed that, compared with the performance of the models constructed with all mRNAs as features (mean MCCs = 0.791), the model performance was improved when modeling with the features identified by our proposed strategy (mean MCCs = 0.800). As for the models with multiscale genomic features, the performance of the models also had been improved to some extent (Figure 4). It indicated that the genomic features selected by our strategy were helpful for the performance of the models. Gene set enrichment analysis revealed that the selected mRNAs and microRNAs were significantly associated with cancers. More specifically, the selected mRNAs were mainly involved in the biological processes of homeostasis in cells, cellular response to drugs, and regulation of hormones. The microRNAs were involved in the processes of gene silencing and inhibition of translation and were mainly associated with the negative regulation of the genes in a set of processes, e.g., the negative regulation of inflammatory response. As for the molecular functions of mRNAs and microRNAs, both of them were highly associated with the binding of genes and proteins, as well as the activity of enzymes. These findings can provide valuable reference for stratifying BCRA patients and exploring the mechanism of the cancer.

In order to illustrate the effectiveness of multiscale genomic features and our proposed strategy for feature selection, we used the ER status of BCRA patients and the tumor stages of KIRC and THCA as the prediction endpoints to evaluate the performance of various models. It is worth noting that the predictive models were data dependent. For different prediction endpoints, the performance of the models will change a lot. The deep optimization of the models would be also conducive to improving the prediction performance of the models, although in this study, the use of a different number of features and default parameters had no significant impact on model performance. As the number of mRNAs is larger than that of miRNAs, if we apply feature selection on the multiple genomic inputs with a simple combination, the top-ranked ones are more likely to be dominated by mRNAs, which can be easily found in our selected feature lists. For leveraging multiscale genomic data, this is a challenging problem that is worth further discussion. Furthermore, we only used the FPKM normalized gene expression values as features in the current study to construct the predictive models, and using different transcript quantification methods applied to represent the gene expression levels, such as RSEM [42], might result in different prediction results.

## 5. Conclusions

In conclusion, compared with a single type of genomic features, multiscale genomic features can provide more information of cancer for constructing the predictive models and help to improve the prediction performance of the models. The integration of multiple genomic features will lead to a sharp increase in the number of features. Effective screening of genomic features closely related to cancer is important for the prediction performance of the models. Our proposed strategy can well identify the genomic features of breast cancer and effectively improve the stratification of breast cancer patients having different estrogen receptor status.

## Figures and Tables

**Table 1:** The KEGG pathways enriched with 289 mRNAs identified by SHAP.

KEGG pathway	*p* value
hsa05120: epithelial cell signaling in *Helicobacter pylori* infection	0.036
hsa05206: microRNAs in cancer	0.036
hsa01100: metabolic pathways	0.034
hsa05200: pathways in cancer	0.026

**Table 2:** The top ten GO terms related to biological processes significantly enriched with 289 mRNAs identified by SHAP.

GO terms	*p* value
GO:0042493~response to drug	*p* < 0.001
GO:0035239~tube morphogenesis	*p* < 0.001
GO:0051093~negative regulation of developmental process	*p* < 0.001
GO:0042592~homeostatic process	*p* < 0.001
GO:0051270~regulation of cellular component movement	*p* < 0.001
GO:0048565~digestive tract development	*p* < 0.001
GO:0048871~multicellular organismal homeostasis	*p* < 0.001
GO:0010817~regulation of hormone levels	*p* < 0.001
GO:0035148~tube formation	0.001
GO:0045595~regulation of cell differentiation	0.002

**Table 3:** The top ten GO terms related to molecular functions significantly enriched with 289 mRNAs identified by SHAP.

GO terms	*p* value
GO:0004716~receptor signaling protein tyrosine kinase activity	*p* < 0.001
GO:0042802~identical protein binding	0.001
GO:0008134~transcription factor binding	0.002
GO:0045502~dynein binding	0.006
GO:0046983~protein dimerization activity	0.011
GO:0019899~enzyme binding	0.017
GO:0016769~transferase activity, transferring nitrogenous groups	0.030
GO:0016772~transferase activity, transferring phosphorus-containing groups	0.035
GO:0044325~ion channel binding	0.039
GO:0019904~protein domain-specific binding	0.046

## Data Availability

The processed data sets and the source code used in our study can be accessed from GitHub (https://github.com/zyrr183/Analysis-of-multiscale-genomic-features).

## Supplementary Materials

Supplementary 1 Supplementary Figure 1. The MCCs achieved by different machine learning algorithms combined with different feature selection methods.

Supplementary 2 Supplementary Figure 2. The MCCs achieved by different machine learning algorithms with different cut-offs of the number of features.

Supplementary 3 Supplementary Figure 3. MCCs for the independent test set by using mRNAs, miRNAs and the combination of mRNAs and miRNAs as features in KIRC.

Supplementary 4 Supplementary Figure 4. MCCs for the independent test set by using mRNAs, miRNAs and the combination of mRNAs and miRNAs as features in THCA.

Supplementary 5 Supplementary Table 1. Three metrics for the independent test set by using mRNAs, microRNAs and the combination of mRNAs and miRNAs as features in BRCA.

Supplementary 6 Supplementary Table 2. Three metrics for the independent test set by using mRNAs, microRNAs and the combination of mRNAs and microRNAs as features in KIRC.

Supplementary 7 Supplementary Table 3. Three metrics for the independent test set by using mRNAs, miRNAs and the combination of mRNAs and miRNAs as features in THCA.

Supplementary 8 Supplementary Table 4. The KEGG pathways significantly enriched with 291 miRNAs identified by SHAP.

Supplementary 9 Supplementary Table 5. The top ten GO terms related to biological processes significantly enriched with 291 miRNAs identified by SHAP.

Supplementary 10 Supplementary Table 6. The GO terms related to molecular functions significantly enriched with 291 miRNAs identified by SHAP.

Supplementary 11 Supplementary Table 7. The MCCs achieved by different machine learning algorithms combined with different feature selection methods in BRCA.

Supplementary 12 Supplementary Table 8. The ACCs achieved by different machine learning algorithms combined with different feature selection methods in BRCA.

Supplementary 13 Supplementary Table 9. The F1 scores achieved by different machine learning algorithms combined with different feature selection methods in BRCA.
