# Supplementary material for: Improving Model Performance on the Stratification of Breast Cancer Patients by Integrating Multiscale Genomic Features
Source: Biomed Res Int. 2020 Aug 25;2020:1475368. doi: 10.1155/2020/1475368 (PMC7471833; doi:10.1155/2020/1475368)
Supplement: Supplementary 8 — Supplementary Table 4. The KEGG pathways significantly enriched with 291 miRNAs identified by SHAP. [file 1475368.f8.docx]

**Supplementary Table 4. The KEGG pathways significantly enriched with 291 microRNAs identified by SHAP.**

| KEGG pathway | *P* Value |
| --- | --- |
| hsa05206: MicroRNAs in cancer | *P* < 0.001 |
