# Supplementary material for: Improving Model Performance on the Stratification of Breast Cancer Patients by Integrating Multiscale Genomic Features
Source: Biomed Res Int. 2020 Aug 25;2020:1475368. doi: 10.1155/2020/1475368 (PMC7471833; doi:10.1155/2020/1475368)
Supplement: Supplementary 9 — Supplementary Table 5. The top ten GO terms related to biological processes significantly enriched with 291 miRNAs identified by SHAP. [file 1475368.f9.docx]

**Supplementary Table 5. The top ten GO terms related to biological processes significantly enriched with 291 microRNAs identified by SHAP.**

| GO term | *P* Value |
| --- | --- |
| GO:0035195~gene silencing by miRNA | *P* < 0.001 |
| GO:0035278~miRNA mediated inhibition of translation | *P* < 0.001 |
| GO:0050728~negative regulation of inflammatory response | *P* < 0.001 |
| GO:0030336~negative regulation of cell migration | *P* < 0.001 |
| GO:1903671~negative regulation of sprouting angiogenesis | *P* < 0.001 |
| GO:0016525~negative regulation of angiogenesis | *P* < 0.001 |
| GO:1903588~negative regulation of blood vessel endothelial cell proliferation involved in sprouting angiogenesis | *P* < 0.001 |
| GO:0010629~negative regulation of gene expression | *P* < 0.001 |
| GO:0045766~positive regulation of angiogenesis | *P* < 0.001 |
| GO:0090051~negative regulation of cell migration involved in sprouting angiogenesis | *P* < 0.001 |
