# Supplementary material for: Improving Model Performance on the Stratification of Breast Cancer Patients by Integrating Multiscale Genomic Features
Source: Biomed Res Int. 2020 Aug 25;2020:1475368. doi: 10.1155/2020/1475368 (PMC7471833; doi:10.1155/2020/1475368)
Supplement: Supplementary 10 — Supplementary Table 6. The GO terms related to molecular functions significantly enriched with 291 miRNAs identified by SHAP. [file 1475368.f10.docx]

**Supplementary Table 6. The GO terms related to molecular functions significantly enriched with 291 microRNAs identified by SHAP.**

| GO term | *P* Value |
| --- | --- |
| GO:0001046~core promoter sequence-specific DNA binding | 0.016 |
| GO:0003727~single-stranded RNA binding | *P* < 0.001 |
| GO:0008035~high-density lipoprotein particle binding | *P* < 0.001 |
| GO:0000993~RNA polymerase II complex binding | *P* < 0.001 |
| GO:1903231~mRNA binding involved in posttranscriptional gene silencing | *P* < 0.001 |
